# The Proto-Oncogene Int6 Is Essential for Neddylation of Cul1 and Cul3 in *Drosophila*

**DOI:** 10.1371/journal.pone.0002239

**Published:** 2008-05-21

**Authors:** Sigal Rencus-Lazar, Yaniv Amir, Junetai Wu, Cheng-Ting Chien, Daniel A. Chamovitz, Daniel Segal

**Affiliations:** 1 Department of Molecular Microbiology, Tel Aviv University, Tel Aviv, Israel; 2 Department of Biotechnology, Tel Aviv University, Tel Aviv, Israel; 3 Institute of Molecular Biology, Academia Sinica, Taipei, Taiwan; 4 Department of Plant Sciences, Tel Aviv University, Tel Aviv, Israel; Universität Heidelberg, Germany

## Abstract

Int6 is a proto-oncogene implicated in various types of cancer, but the mechanisms underlying its activity are not clear. *Int6* encodes a subunit of the eukaryotic translation initiation factor 3, and interacts with two related complexes, the proteasome, whose activity is regulated by Int6 in *S. pombe*, and the COP9 signalosome. The COP9 signalosome regulates the activity of Cullin-Ring Ubiquitin Ligases via deneddylation of their cullin subunit. We report here the generation and analysis of two *Drosophila* mutants in *Int6*. The mutants are lethal demonstrating that *Int6* is an essential gene. The mutant larvae accumulate high levels of non-neddylated Cul1, suggesting that Int6 is a positive regulator of cullin neddylation. Overexpression in *Int6* in cell culture leads to accumulation of neddylated cullins, further supporting a positive role for Int6 in regulating neddylation. Thus Int6 and the COP9 signalosome play opposing roles in regulation of cullin neddylation.

## Introduction

Int6/eIF3e is a conserved onco-protein encoded by a gene initially identified as a preferred integration site of the mouse mammary tumor virus [1]. Integration of the virus into *Int6* results in expression of a C-terminally truncated version of Int6, thereby causing tumorigenic transformation [2], [3]. Silencing of *Int6* in HeLa cells results in mitotic defects [4].

Int6 is a common interactor of three related protein complexes: the eukaryotic translation Initiation Factor 3 (eIF3), the proteasome and the COP9 signalosome (CSN) [5]. Int6 has been identified as a subunit of eIF3 [6]. In *S. pombe*, Int6 is essential for both assembly and function of the proteasome [7]. As a result, degradation of several proteasome substrates is impaired in *S. pombe* mutated for the *Int6* homolog, *yin6* [7]. It is not clear whether this regulation of the proteasome by Int6 is also conserved in other organisms, since evidence for such a regulation was not found in mammalian cells or in *Arabidopsis* [4], [8].

The CSN is an eight-subunit protein complex involved in regulation of protein degradation. The CSN has been shown to regulate ubiquitination via the Cullin-Ring ubiquitin Ligases (CRLs). The CSN removes the Nedd8 modification from the cullin subunit of the CRL, resulting both in inactivation of the CRL as a ubiquitin ligase [9], [10] and in stabilization of the cullin subunit in some organisms [11]–[13]. On the other hand, the CSN is also essential for maintaining the stability of the substrate adaptor subunit of CRLs, thereby promoting their ubiquitin ligase activity [14]. The CSN therefore regulates both the stability and the activity of cullin-based E3 ubiquitin ligases.

Int6 co-purifies with the CSN from *Arabidopsis* [15] and interacts with several CSN subunits both in plants and in mammalian cells [5], [16], but the significance of this interaction is not yet clear. To elucidate the roles of Int6 in the context of a multi-cellular organism, we analyzed two mutants in *Drosophila Int6*, demonstrating it to be an essential gene for both organism and cell viability. While we did not detect any disruption in CSN assembly or function in *Int6* mutants, we found a decrease in neddylation of both Cul1 and Cul3 in the absence of Int6. Our findings also suggest that Int6 is essential for the degradation of cullin substrates. Consistently, overexpression of Int6 in cell culture resulted in elevated neddylation of these two cullins. We thus show here a novel role for Int6 as a positive regulator of cullin neddylation.

## Methods

### *D. melanogaster* maintenance and hybrid dysgenesis

Strains were maintained and crosses were conducted on standard cornmeal-molasses medium at 25°C. Oregon-R served as the wild-type control. The *Nedd8^172^*, *Cul1^EX^* [17], *CSN5^null^* [18] *Cul3^06430^*, *Cul3^d577^* [19] and *Rpn10* [20] mutations have been previously described. Description of balancer chromosomes and markers can be found in FlyBase [21] (http://flybase.bio.indiana.edu/). The CyO and TM3 Ser balancers that carry the act-GFP transgene are described elsewhere [22].

The P element in Bloomington strain #11748 [21] is inserted within the coding sequence of *Int6* at base 204, where 1 is the 1st base of the start codon. Analysis of putative excision lines was performed with primers derived from *Int6* sequences upstream to the P element insertion (5′-ATGGCCAATTTCGATCTGACACG-3′), corresponding to nucleotides 1 to 23, and downstream of the P insertion site (5′-CTAGTAGTACTTCCAGGAGTCGG-3′) corresponding to *Int6* gene sequence 1462 to 1484. The P element in the 11748 strain carries the dominant *ry*+ eye color marker. Crosses of females of the 11748 strain and males of a strain carrying the 2–3 stable source of transposase (Bloomington Stock Center #1610) were conducted. Dysgenic *P{ry+t7.2 = PZ}Int6[10547] ry506/TMS*, *P{ry[+t7.2] = Delta2-3}99B* male progeny, in whose germ line the *P{ry+t7.2 = PZ}Int6[10547]* P element was mobilized, were crossed to females of the 11748 strain. Offspring (F2) that did not carry the transposase source were screened for males with rosy eyes indicative of loss of the *ry*+ marker caused by excision the P element from 11748. Individual putative excision males were crossed to females of the 11748 strain to establish balanced lines. Lines were established from 57 independent putative excision flies, 6 of which were homozygous viable.

### Heat shock treatment

#### Generation of germ-line clones

flies of the desired cross were allowed to lay eggs for twenty-four hours in small tubes containing standard cornmeal-molasses medium. The larvae were heat-shocked at the 2^nd^ and 3^rd^ instar stages. Heat shock was administered by putting the vials with the larvae in a 37°C water bath for 1.5 hours. Heat shock was performed twice a day on days 3 and 4 after egg laying. After the heat treatment the vials were returned to 25°C.

#### Generation of wing and eye disc clones

flies of the desired cross were allowed to lay eggs for twenty-four hours in tubes containing standard medium. Heat shock was administered by putting the vials with the larvae in a 37°C water bath for 1.5 hours 5 days after performing the cross. The larvae were dissected two days (∼44 hours) after the heat shock treatment.

### Molecular and biochemical procedures

#### Quantitative reverse transcription PCR

RNA was extracted from ∼1000 Oregon-R (wt) and ∼2500 *Int6^173-1^* 1st instar larvae with TRIzol reagent (Invitrogen). cDNA synthesis was carried out using Superscript II (Invitrogen).

Four qPCR primer pairs were designed to generate intron-spanning products using Primer Express Version 1.5 software:

Primer name  Sequence
Cul1-RT5  GTGCTGACCTCCAGCGACA
Cul1-RT6  GGTTGATGTTAATTCGGCGC
Rp49-forward  TAAGCTGTCGCACAAATGGC
Rp49-reverse  ACCGATGTTGGGCATCAGATA

Quantitative reverse transcription polymerase chain reaction (qRT-PCR) analysis was performed on an ABI Prism® 7000 Sequence Detection System (Applied Biosystems) using SYBR Green® I chemistry. Samples were in triplicates and normalized for RNA levels based on *rp49* expression. Analysis was performed with the ABI Prism 7000 SDS software RQ study Application v1.1 using the Δ-ΔCt method, which determines fold changes in gene expression relative to a comparative sample.

### SDS-PAGE and Immuno-blot

Protein samples were mixed with 1∶1 volume of Laemmli sample buffer (BioRad), were separated on a 10% poly-acrylamide gel using Mighty Small System (Hoefer). Proteins were transferred onto a PVDF membrane (Millipore), pre-washed in methanol, in transfer buffer. The membrane was then blocked for 1 hour in blocking solution (5% milk powder, 0.02% sodium-azide in 1xTBS), and then incubated in the primary antibody (see below) diluted in blocking solution. The membrane was then washed 3 times for 15 minutes in TTBS (0.1% Tween-20 in 1xTBS), incubated for 30 minutes in the secondary antibody and washed 3 times for 10 minutes in TTBS. The membrane was developed using EZ-ECL (Biological industries), according to the manufacturer's instructions, and exposed to Fuji Medical X-Ray Film for up to 5 minutes. Films were developed using Kodak X-OMAT 2000.

Primary antibodies used for immuno-blot analysis were anti-Cul1 (1∶250; Zymed), anti-actin (1∶5000; MP Biomedicals), anti-Cul3 (1∶1000), anti-Nedd8 (1∶1000), anti-Ubiquitin (1∶7500; Affinity), anti-cyclin B (1∶3000) [24], anti-CSN5 (1∶10000) [25], anti-CSN7 (1∶10000) [25] and anti-HA (1∶2500; Covance). Secondary antibodies were Horse radish peroxidase-conjugated goat-anti-rabbit (1∶10000; Santa Cruz), and Horse radish peroxidase-conjugated goat-anti-mouse (1∶10000; Jackson Immuno-research).

### Signal quantification

Immuno blots displaying a signal within the ECL linear response were scanned using an ImageScanner (Amersham Pharmacia Biotech) and bands were quantified using the ImageMaster 1D Image Analysis Software (Amersham Pharmacia Biotech).

### Cell culture methods

Schneider's 2R+ cells were grown in flasks at 27°C in Schneider's *Drosophila* medium with L-Glutamine (Biological Industries) containing 10% Fetal Calf Serum (pre-inactivated for 1 hour at 60°C; Biological Industries), 100 units/ml Penicillin and 0.1 mg/ml Streptomycin-Sulfate (Biological Industries). Cells were transferred 1∶4 every 3–4 days. For trasnfection, four days after a regular 1∶4 transfer, cells were transferred 1∶3 into each well of a 6-well plate. On the following day 3.5 µg of each plasmid (∼1.5 µg/µl) were added to serum- and antibiotics-free medium to a total volume of 100 µl. In parallel, 10 µl ESCORT IV Transfection Reagent (Sigma) were added to 90 µl serum- and antibiotics-free medium. The diluted DNA was then added to the diluted transfection reagent and the DNA-liposome complexes were allowed to form for 1 hour at room temperature. The cells (50–70% confluent) were washed 4 times in serum- and antibiotics-free medium and 800 µl serum- and antibiotics-free medium were then added to each well. 200 µl of the DNA-ESCORT mixture were then added to each well in a dropwise manner. The cells were grown at 27°C for 23 hours, after which the serum- and antibiotics-free medium was changed to serum- and antibiotics-containing medium. Cells were grown for additional 8 hours for a total of 31 hours, and the cells in each well were then resuspended in the 1 ml medium and transferred into a tube. Cells were harvested by centrifugation for 3 min at 2000 rpm. The cells pellet was frozen at −80°C. Over expression of the transfected gene was verified by SDS-PAGE, followed by immunoblot analysis.

## Results

### *Int6* is an essential gene

A strain was obtained (Bloomington #11748) that contains a P element located in the *Int6* open reading frame (*P{PZ}Int6^10547^*) [21]. PCR and sequence analysis confirmed that the P element in the genome of the 11748 strain is inserted in the second exon of *Int6*, 204 bp downstream of the 1^st^ ATG codon (Figure S1).

The chromosome carrying the insert in *Int6* is homozygous lethal. To verify that the lethality of the *P{PZ}Int6^10547^*-carrying chromosome is due to the P insertion in *Int6*, the P element was mobilized through a hybrid dysgenesis protocol of crosses. Six homozygous viable excision lines were obtained and sequence analysis confirmed the excision of the P element and restoration of the normal *Int6* gene sequence. Thus, *Int6* is essential for *Drosophila* development.

Homozygous lethal excision lines were also obtained in the hybrid dysgenesis. PCR and sequence analysis of one of these lines, termed *Int6^173-1^*, indicated a deletion of base pairs 204 to 952 in the *Int6* gene, where 1 is defined as the first nucleotide in the start codon (Figure S1).

**Figure 1:**
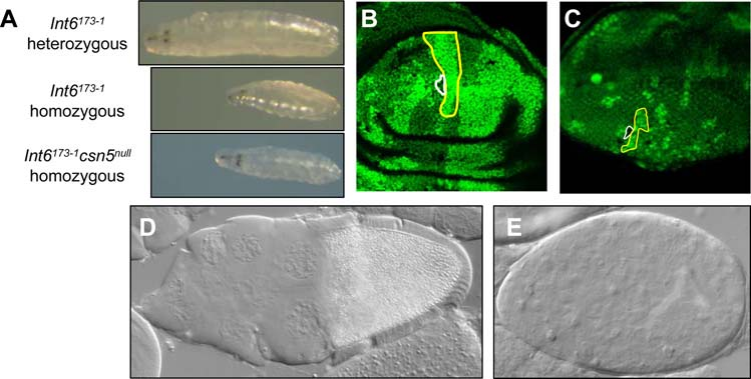
*Int6* mutant phenotypes. A. Larvae heterozygous (upper panel) and homozygous (middle panel) for the *Int6^173-1^* allele are shown. Larvae homozygous for the *P{PZ}Int6^10547^* allele display an identical phenotype (not shown). *Int6^173-1^-csn5^null^* double mutant larvae are phenotypically identical to *Int6^173-1^* single mutants (lower panel). B,C. Clones homozygous for the *P{PZ}Int6^10547^* mutation display growth/viability defects. *Int6* clones in wing (B) and eye (C) disc (for example, outlined in white), are shown compared to the markedly larger GFP/GFP twin spots (for example, outlined in yellow); D–E. Egg chambers from germ line clones of a positive control, which developed into embryos (D) and of the *P{PZ}Int6^10547^* mutation, arrested at stage 5–6 of oogenesis (E). Somatic and germ line clones of the *Int6^173-1^* deletion result in the same phenotypes as the *P{PZ}Int6^10547^* mutation (not shown).

Both the *Int6^173-1^* deletion and the P insertional mutation result in essentially identical phenotypes. Homozygous larvae for either mutation are small, compared to their heterozygous siblings, and die within 3–4 days after hatching while still at the 1^st^ larval instar stage (Figure 1A, two upper panels, and data not shown), whereas their heterozygous siblings have progressed by then to the 3^rd^ instar. The *Int6* mutant larvae do not display any other obvious morphological abnormalities. Larvae heteroallelic for the two *Int6* mutations show the same phenotype.

To examine the phenotype of the lack of Int6 at the cellular level, we generated both somatic and germ-line clones homozygous for either *Int6* mutation. As shown in Figure 1B,C, *Int6*-homozygous clones induced in both the wing and eye imaginal discs of several dozen individuals were found to be much smaller than their adjacent non-mutant twin spots, indicating that *Int6* is essential for viability and/or proliferation of the disc cells. By the adult stage, the *Int6*-homozygous clones were apparently eliminated as both adult eyes and wings did not show any aberrant phenotype (not shown).

To examine the roles of Int6 when both zygotic and maternally contributed Int6 are absent, germ line clones homozygous for either *Int6* mutation were generated [26]. While germ-line clones of the control *Int6^+^* chromosome contained fully developed egg chambers (Figure 1D), that gave rise to normal embryos, germ line clones homozygous for either *Int6* mutation arrested at stage 5–6 of oogenesis (Figure 1E). This suggests that as in somatic cells, Int6 is required for proliferation or viability of female germ cells, and thereby for proper oogenesis.

### Int6 is a negative regulator of Cul1 protein levels

**Figure 2:**
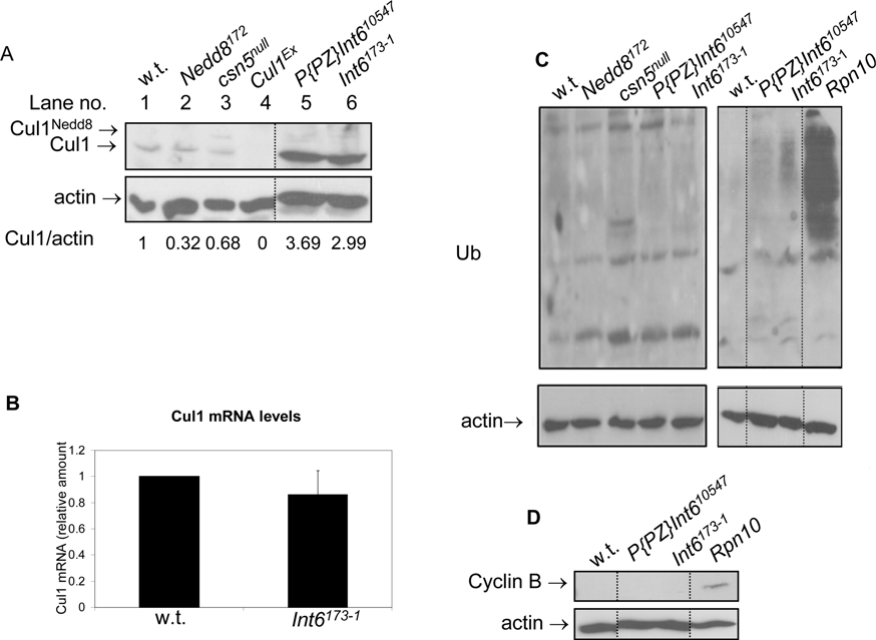
Int6 is a negative regulator of Cul1 protein levels. A. Immuno-blot analysis using anti-Cul1 antibodies performed on protein samples extracted from homozygous larvae of the indicated genotypes. B. Real-time PCR analysis of *Cul1* mRNA levels in wild type and *Int6^173-1^* mutants at the same ages as in A. Transcript levels in the mutant are presented in relation to wt. Error bars are the standard deviation of three repeats. C–D. Immuno-blot analysis using anti-ubiquitin (C) and anti-cyclin B (D) antibodies performed on protein samples extracted from 1^st^ instar larvae of the indicated genotypes. *Rpn10* mutants were used as a positive control for proteasome malfunction. Samples were run on a single gel and lane order was rearranged for the figure as indicated by the dotted lines. The blot in panel A was repeated with similar results two times.

Int6 physically interacts with several CSN subunits in both Arabidopsis [16] and mammalian cell culture [5]. These interactions take place in *Drosophila* as well (see below and data not shown), but do not serve to regulate the assembly of the CSN (Figure S2). To further explore the interaction between Int6 and the CSN, we examined whether Int6 regulates the deneddylation function of the CSN by comparing the levels of non-neddylated and neddylated Cul1 in larvae of various genotypes. While, as expected, neddylated Cul1 accumulated in *csn5^null^* larvae, compared to wild type control (Figure 2A, compare lanes 1 and 3) [27], no accumulation of neddylated-Cul1 was observed in either *Int6* mutant, 72 hours AED (Figure 2A, compare lanes 1 and 5,6). This result indicates that *Int6* is not essential for CSN deneddylation activity. Surprisingly though, we found high accumulation of Cul1 in its non-neddylated form in both *P{PZ}Int6^10547^*, the P-element insertion mutant of *Int6*, and in *Int6^173-1^*, the *Int6* deletion mutant (Figure 2A, compare lanes 1 and 5,6). This result was confirmed for each *Int6* mutant allele by at least one additional biological repeat. The accumulation of non-neddylated Cul1 in two different *Int6* mutants demonstrates that Int6 is a negative regulator of Cul1 levels in *Drosophila*. The high level of non-neddylated Cul1 in Int6 mutants is not a result of elevated RNA levels since the levels of *Cul1* mRNA in the *Int6^173-1^* mutants are not significantly different from the wild type control (Figure 2B). Thus, Int6 negatively regulates Cul1 in a post-transcriptional mechanism.

Another possible mechanism for the regulation of Cul1 levels by Int6 would be via regulation of proteasome function. As Int6 is essential for the function of the proteasome in *S. pombe* [7], it is possible that proteasome substrates, one of which may be Cul1 [28], accumulate in *Drosophila Int6* mutants. To test this possibility, we examined the general function of the proteasome in the two *Int6* mutants by monitoring the levels of ubiquitinated proteins. No accumulation of ubiquitinated proteins was detected in either *Int6* mutant, nor in *csn5^null^* or *nedd8* mutants (Figure 2C, left panel). High accumulation of ubiquitinated proteins was detected in larvae mutant for the proteasome regulatory subunit *Rpn10*, used here as a positive control (Figure 2C, right panel, compare lanes 1 and 4), while again, no similar accumulation was detected in either *Int6* mutant (Figure 2C, compare lanes 1 and 2,3). To further assay proteasome function in these mutants, we monitored the levels of cyclin B, a specific proteasome substrate which accumulates in *Int6* mutants in *S. pombe* [7]. While cyclin B accumulated in *Rpn10* mutants (Figure 2D, compare lanes 1 and 4), it did not in either *Int6* mutant (Figure 2D, compare lanes 1 and 2,3). Taken together, these results clearly indicate that the accumulation of Cul1 in the absence of Int6 is not a result of a reduced proteasome activity.

### Int6 is a positive regulator of Cul1 neddylation

**Figure 3:**
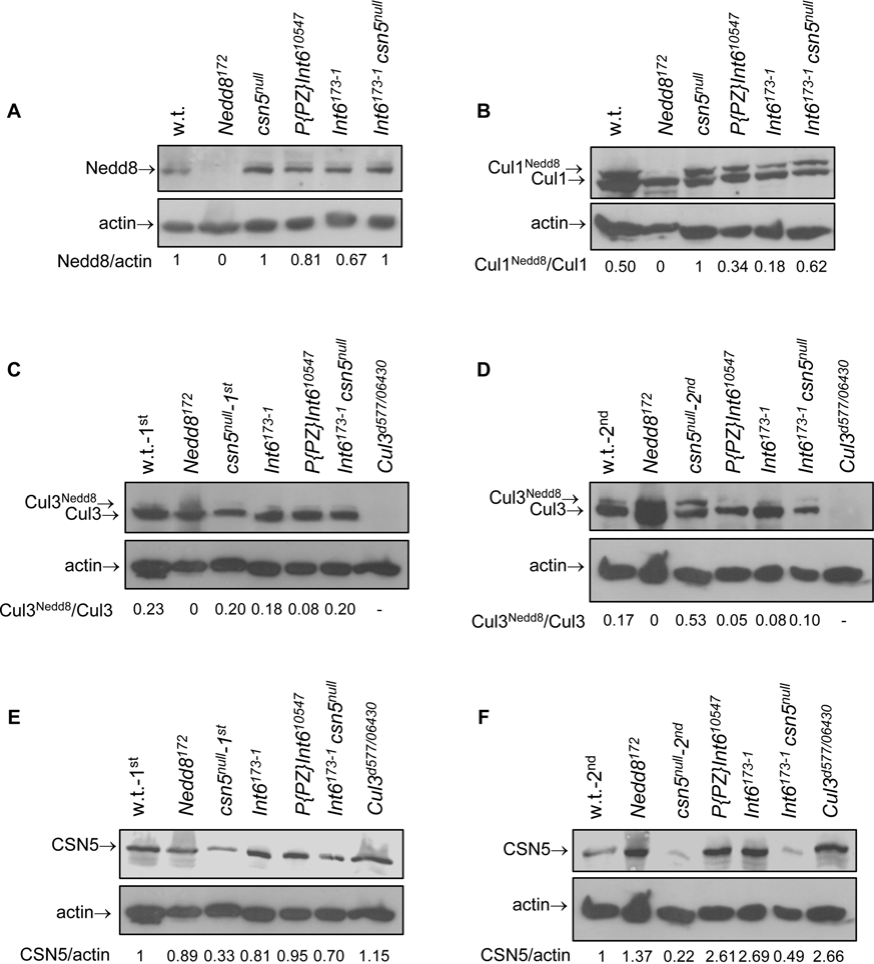
Int6 is essential for neddylation of Cul1 and Cul3 and is a negative regulator of CSN5 levels. Immno-blot analysis using anti-Nedd8 (A), anti-Cul1 (B), anti-Cul3 (C,D) or anti-CSN5 (E,F) antibodies performed on protein samples extracted 48 (A–C,E) or 72 (D,F) hours after egg deposit of the indicated genotypes. In the absence of Int6, both overall neddylation of cullins (A), and specifically the neddylation of Cul1 (B) and Cul3 (D), are reduced. CSN5 is accumulated in the absence of either Int6 or Cul3 (F).

Neddylation of *Drosophila* Cul1 results in its destabilization [12]. Another possible explanation for the accumulation of high levels of non-neddylated Cul1 in *Int6* mutants would therefore be reduced Cul1 neddylation in the absence of Int6. To test this possibility we used anti-Nedd8 antibodies, and indeed found reduced intensity of ∼90 kDa bands (the expected size of neddylated cullins) in both *Int6* mutants (Figure 3A, compare lanes 1 and 4, 5). If this reduced neddylation is the cause of the accumulation of Cul1 in the absence of Int6, accumulation of non-neddylated Cul1 in the *Int6* mutants should be preceded by reduced Cul1 neddylation. To examine this possibility, we examined the ratio of neddylated∶non-neddylated Cul1 in 48-hours-old *Int6* mutants. As expected, this ratio was two-fold higher in *csn5^null^* mutants, compared to the wild type control (Figure 3B, compare lanes 1 and 3). Yet, the ratio of neddylated∶non-neddylated Cul1 was significantly reduced in both *Int6* mutants (Figure 3B, compare lanes 1 and 4, 5, and biological repeats not shown), indicating that Cul1 neddylation is indeed reduced in the absence of Int6.

Since the neddylated: non-neddylated Cul1 ratio is higher than normal in *csn5^null^* mutants and lower than normal in *Int6* mutants, we reasoned that the *csn5^null^* mutation should rescue the reduced Cul1 neddylation in *Int6* mutants. Indeed, the level of neddylated Cul1 was rescued in *Int6^173-1^-csn5^null^* double mutant larvae, compared to each single mutant (Figure 3B, compare lanes 3,5 and 6). This result further implicates Int6 in regulation of neddylation of Cul1, since in the absence of both Int6 and CSN5, both neddylation and deneddylation are reduced, resulting in an intermediate neddylated: non-neddylated Cul1 ratio. Phenotypically, the double mutant *Int6^173-1^-csn5^null^* homozygous larvae are very similar to the *Int6^173-1^* single mutants (Figure 1A, lower panel), suggesting that the phenotypes of the *Int6* mutants are not solely due to lack of Cul1 neddylation. Taken together, these results indicate that Cul1 neddylation is reduced in the absence of Int6, and as a result Cul1 accumulates in its non-neddylated form.

### Int6 is a positive regulator of Cul3 neddylation

We next examined whether Int6 regulates the neddylation of additional cullins. Immuno-blot analysis using anti-Cul3 antibodies revealed a significant decrease in Cul3 neddylation in *P{PZ}Int6^10547^* homozygous larvae, compared to the wild type control (Figure 3C, compare lanes 1 and 5), but only a slight decrease in *Int6^173-1^* homozygous larvae (Figure 3C, compare lanes 1 and 4). It should be noted that 48 hours old *csn5^null^* larvae did not show any increase in neddylated: non-neddylated Cul3 ratio (Figure 3C, compare lanes 1 and 3), perhaps due to the persistence of maternally deposited CSN5 at this stage (Figure 3E, lane 3).

To further examine whether Int6 regulates the neddylation of Cul3, we performed the same immuno-blot analysis on 72-hours-old *csn5^null^* and *Int6* mutants. Second instar *csn5^null^* larvae displayed the expected increase in the ratio of neddylated∶non-neddylated Cul3, compared to the wild type control, due to reduced deneddylation activity of the CSN (Figure 3D, compare lanes 1 and 3). Decreased Cul3 neddylation was found in 72-hours-old larvae of both *Int6* mutants (Figure 3D, compare lanes 1 and 4, 5, and biological repeats not shown), demonstrating that Int6 is essential for neddylation of Cul3, as well as of Cul1. Nevertheless, the *csn5^null^* mutation did not rescue the reduced Cul3 neddylation caused by the lack of Int6 since Cul3 neddylation in the *Int6^173-1^-csn5^null^* double homozygous larvae is very similar to that of the *Int6^173-1^* single mutant (Figure 3D, compare lanes 5 and 6).

### Int6 is a negative regulator of CSN5 levels

**Figure 4:**
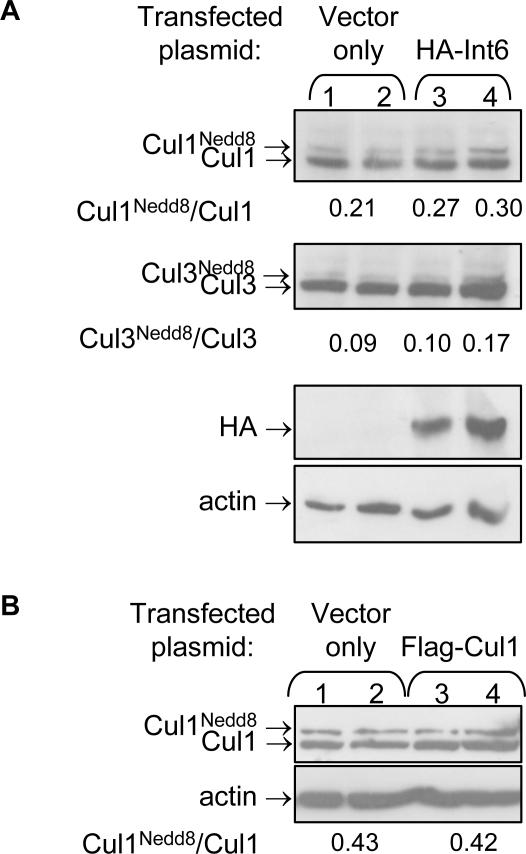
Int6 promotes neddylation of Cul1 and Cul3. A,B. Immuno-blot analysis using anti-Cul1 (A,B) and anti-Cul3 (A) antibodies performed on protein samples extracted from Schneider's 2R+ cells transfected with either HA-Int6 (A) or Flag-Cul1 (B). Over-expression of HA-Int6 results in a specific elevation of neddylated Cul1 and Cul3 (A), though neddylation of the latter is increased only in the presence of high levels of Int6. This elevated neddylation is not a result of mere high cullin levels, since over-expression of Flag-Cul1 results in an equal elevation of Cul1 and neddylated Cul1 (B).

The levels of *csn5* mRNA were recently shown to increase as a result of the expression of a dominant negative version of Int6 in transgenic mice [29]. An equivalent increase in CSN5 protein levels in *Drosophila Int6* mutants might explain the reduced neddylated: non-neddylated cullin ratio we found in both *Int6* mutants (Figure 3A, B, D), since elevated CSN5 levels would result in increased deneddylation activity of the CSN. However, this possible explanation does not apply for the decreased neddylation of Cul3 in both *Int6* mutants since it is not rescued by the *csn5^null^* mutation (Figure 4D). We found that the levels of CSN5 indeed increase in both *Int6* mutants, but only in 72 hours old larvae (Figure 3E,F, compare lanes 1 and 4,5). Since Cul1 neddylation is already low in 48 hours old larvae (Figure 3B), the accumulation of CSN5 in the absence of Int6 can not account for the reduction in the neddylation of either Cul1 or Cul3. It should be noted, that similar to previously reported results in *Arabidopsis* [13], *Drosophila* CSN5 over-accumulates in the absence of Cul3 (Figure 3F, compare lanes 1 and 7).

### Int6 is sufficient for promoting neddylation

Having demonstrated that Int6 is essential for cullin neddylation, we examined whether it is also sufficient for promoting neddylation. Over-expression of HA-tagged Int6 in *Drosophila* Schneider cell culture led to specific elevation of the neddylated form of both Cul1 and Cul3 (Figure 4A, compare lanes 1, 2 and 3, 4). While the levels of non-neddylated Cullins were not affected by the overexpression of Int6, the neddylated form of Cul1 was increased by ∼40%, and the neddylated form of Cul3 was increased by 89%. As opposed to Cul1, Cul3 neddylation was elevated only in the presence of large amounts of over-expressed Int6 (Figure 4A, compare lanes 3 and 4). Since neddylation of cullins also destabilizes them [12], the elevated neddylation of both cullins in the presence of over-expressed Int6 could be an indirect result. In this scenario, over-expressed Int6 causes an elevation in cullin levels, and as a result excess cullins are tagged by Nedd8 for degradation. To examine this possibility we over-expressed Flag-tagged Cul1 in Schneider cell culture. Over-expression of Flag-Cul1 resulted in an equal increase of both neddylated and non-neddylated Cul1 (Figure 4B, compare lanes 1,2 and 3,4), indicating that mere rise of Cul1 levels does not result in elevated neddylation. We therefore conclude that Int6 is sufficient for promoting neddylation of both Cul1 and Cul3.

## Discussion

This work aimed at analyzing the roles of Int6 during *Drosophila* development in the context of its interactions with the CSN. The CSN has been shown to de-neddylate cullins, thereby regulating degradation of their substrates by the ubiquitin-proteasome pathway. We show here, for the first time, that Int6 takes part in the regulation of this process by promoting neddylation of Cul1 and Cul3. This was clearly shown in each of the two different *Int6* mutants, which provide internal repeats for all experiments. Therefore, Int6 and the CSN play opposing roles in regulation of the neddylation process.

### *Int6* is an essential gene in *Drosophila*

To examine the roles of Int6 in the context of a multi-cellular organism, we analyzed two mutants in *Drosophila Int6*. Both the deletion and a P element insertional mutations described in this study are homozygous lethal at the 1^st^ instar larval stage. In addition, *Int6* mutant clones in the wing disc, the eye disc, or the germ line, were very small compared to their non-mutant twin-spots, and were eliminated from adult tissue. Int6 is thus likely an essential general cellular regulator in *Drosophila*.

In spite of numerous attempts, though, we were not successful in generating effective antibodies against *Drosophila* Int6, nor were previously reported antibodies [30] reactive in our hands, thereby precluding a more complete biochemical analysis of Int6.

### Int6 promotes neddylation of Cul1 and Cul3

We show here a novel role for Int6 as a positive regulator of cullin neddylation. Although the mechanism by which Int6 regulates cullin neddylation is as yet to be established, it likely does not involve direct interaction of Int6 with Cullins as we could find no evidence for such an interaction by immunoprecipitation (not shown). This novel role of Int6 might shed some more light on the roles Int6 plays in cancer development. As we show here (Figures 3), the absence of Int6 results in reduced cullin neddylation, as well as in the accumulation of CSN5. The levels of CSN5 are also increased in the absence of Cul3 in both *Arabidopsis* [13] and *Drosophila* (Figure 3), which may suggest it to be a substrate of Cul3 in both organisms. We therefore propose that Int6 regulates the degradation of at least certain cullin substrates via regulation of cullin neddylation. The abrogated degradation of various cullin substrates in the absence of Int6 may play a key role in the development of cancer. To test this hypothesis, the levels of *bona fide* CRL substrates should be examined in *Int6* mutants. In addition, over-expression of a C-terminally truncated version of Int6 was shown to result in tumorigenic transformation [3], [29]. It will therefore be interesting to examine the effect of over-expression of this truncated Int6 on neddylation of Cul1 and Cul3.

It should be noted that there are several differences between Cul1 and Cul3 with respect to regulation of their neddylation by Int6. The reduced neddylation of Cul1 leads to high accumulation of non-neddylated Cul1 in both *Int6* mutants and is rescued by a mutation in *csn5* (Figure 2,3). However, reduced neddylation of Cul3 is detected later than that of Cul1 (Figure 3), and therefore non-neddylated Cul3 does not accumulate prior to the larvae death. In addition, the reduced neddylation of Cul3 is not rescued by a *csn5* mutation. Consistently, unlike Cul1, neddylation of Cul3 is promoted in cell culture only in the presence of high levels of over-expressed Int6 (Figure 4). We also found a difference in deneddylation of Cul1 and Cul3 by CSN5, since deneddylation of Cul1 is decreased earlier than that of Cul3 in *csn5^null^* mutants. These differences may imply that the regulation of neddylation of Cul1 and Cul3 is not identical. It is possible that neddylation of Cul3 is regulated by other factors, in addition to Int6 and CSN5, and therefore the effect of the latter two on the status of Cul3 neddylation is smaller than their effect on Cul1.

### Int6 is not essential for proteasome function in *Drosophila*

In *S. pombe*, Int6 regulates the function of the proteasome by promoting the entry of the proteasome regulatory subunit Rpn5 into the nucleus [7]. As a result, *S. pombe* mutants lacking Int6 show both a reduction in general proteasome function and accumulation of specific proteasome substrates, Cut2/securin and Cdc13/cyclin B. In contrast, our results show that in the absence of *Drosophila* Int6 both general proteasome function and the degradation of a specific proteasome substrate, cyclin B, are normal, indicating that Int6 is not essential for proteasome activity in the fly (Figure 2). The possibility that Int6 regulates the degradation of a subset of proteasome substrates, and that unlike the situation in *S. pombe*, this subset does not include cyclin B, remains to be tested. Nevertheless, taken together, our results and the findings that Int6 is not essential for degradation of securin and cyclin B in HeLa cells [4] and is not a positive regulator of proteasome function in *Arabidopsis* (A. Yahalom and D.A. Chamovitz, unpublished) indicate that regulation of proteasome function by Int6 is not conserved in higher organisms. It should also be noted, that unlike *Int6* in *S. pombe* [31], [32], we show here that *Drosophila Int6* is an essential gene. It is therefore plausible that the mechanisms underlying Int6 function in both organisms are different as well.

Int6 is a multi-functional protein involved in multiple processes. It has been implicated in regulation of translation [32], [33], mitosis [4] and proteasome regulation in *S. pombe* [7]. Here we show a novel role for Int6 as a positive regulator of cullin neddylation. Further work will be needed to assemble all the existing data into a unified picture of the roles of Int6, both in normal cellular function and in cancer.

## Supporting Information

Figure S1 (2.35 MB TIF)

Figure S2 (2.36 MB TIF)
