# Supplementary figures and images for: The Proto-Oncogene Int6 Is Essential for Neddylation of Cul1 and Cul3 in Drosophila
Source: PLoS One. 2008 May 21;3(5):e2239. doi: 10.1371/journal.pone.0002239 (PMC2375110; doi:10.1371/journal.pone.0002239)

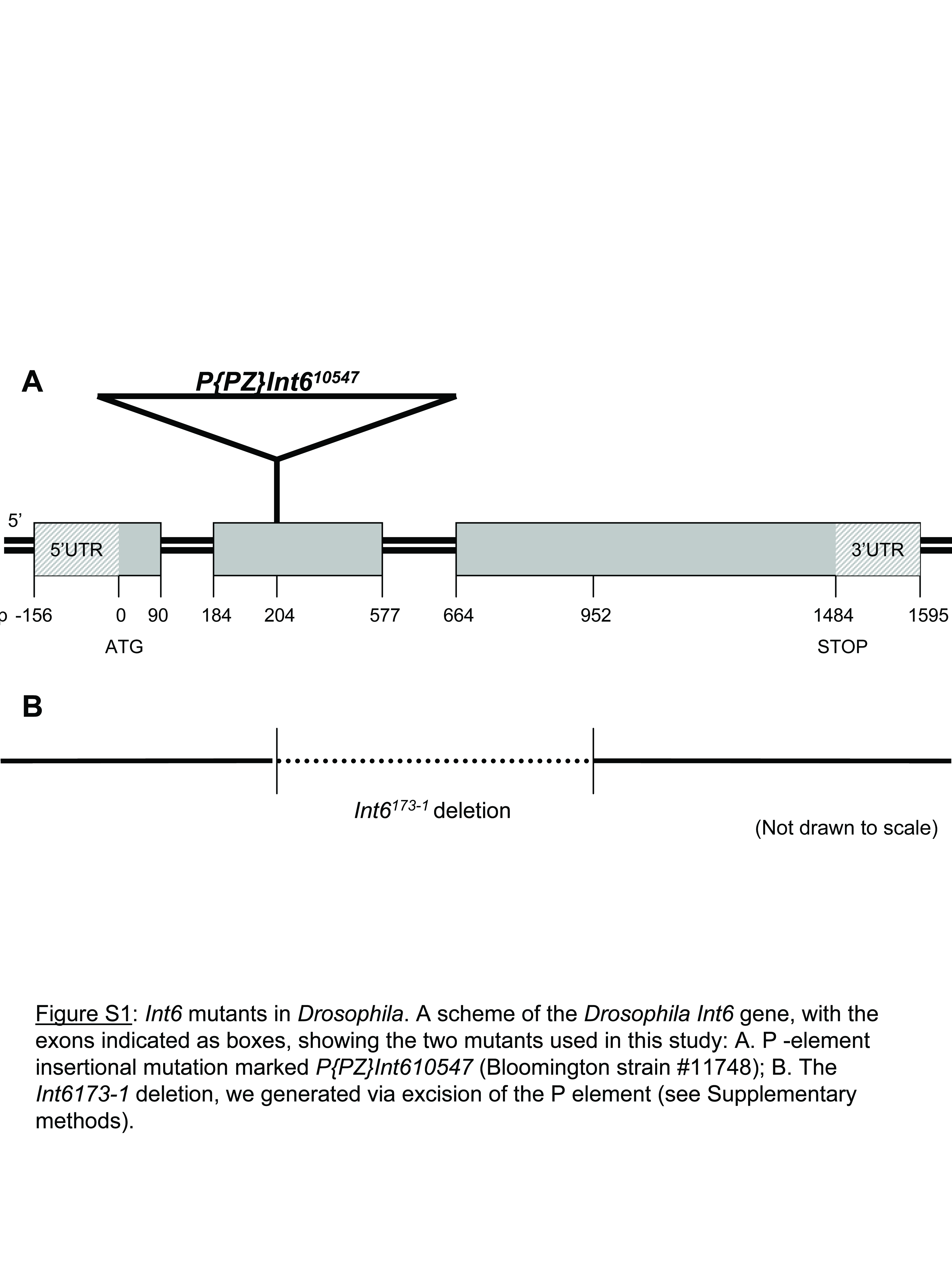

Supplement: Figure S1 — (2.35 MB TIF) [file pone.0002239.s001.tif]

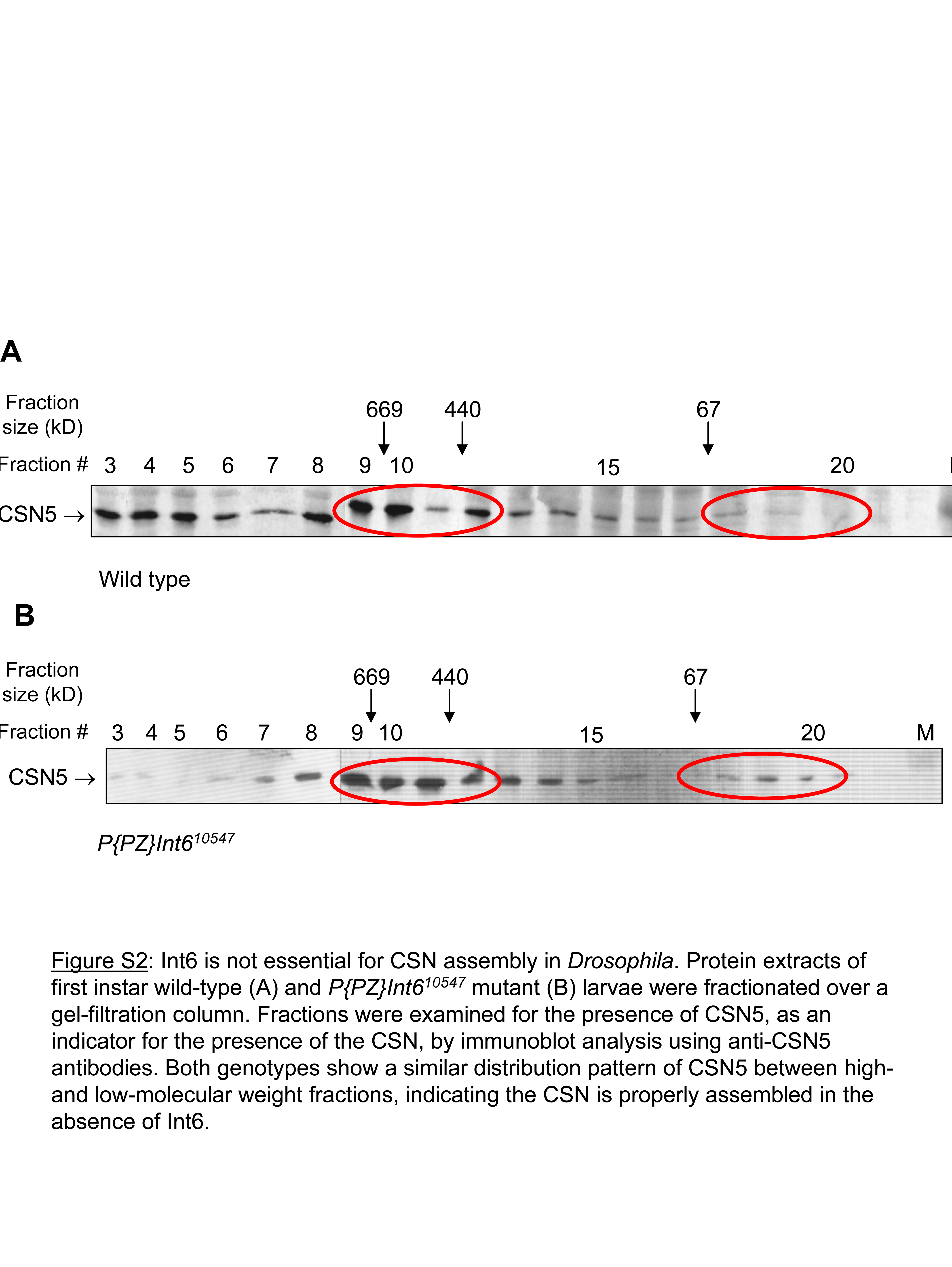

Supplement: Figure S2 — (2.36 MB TIF) [file pone.0002239.s002.tif]
